# *Rasamsonia argillacea* brain abscess in a lung transplant recipient: an unexpected infection by an unusual fungal pathogen

**DOI:** 10.1016/j.mmcr.2025.100727

**Published:** 2025-09-16

**Authors:** Raphaël Schils, Michaël Desjardins, Charles Poirier, Hugo Chapdelaine, Marie-Pierre Fournier Gosselin, Philippe J. Dufresne, Me-Linh Luong

**Affiliations:** a Division of Infectious Disease, Department of Medicine, University of Liege, Liege, Belgium; b Division of Infectious Disease and Microbiology, Department of Medicine, University of Montreal Hospital Center, Montreal, Quebec, Canada; c Department of Microbiology, Infectious Diseases and Immunology, Université de Montréal, Canada; d Division of Respirology, Department of Medicine, University of Montreal Hospital Center, Montreal, Quebec, Canada; e Division of Allergy and Clinical Immunology, Department of Medicine, University of Montreal Hospital Center, Montreal, Quebec, Canada; f Department of Neurosurgery, University of Montreal Hospital Center, Montreal, Quebec, Canada; g Laboratoire de santé publique du Québec, Institut national de santé publique du Québec, Sainte-Anne-de-Bellevue, Québec, Canada

**Keywords:** *Rasamsonia argillacea*, Chronic granulomatous disease, Lung transplant

## Abstract

Fungal infections are an important cause of morbidity and mortality after lung transplantation. While Aspergillus sp. is the most common implicated organism, rare and emerging molds are increasingly reported. We report a case of *Rasamsonia argillacea* (formerly *Geosmithia argillacea*) brain abscess in a lung transplant recipient and highlight the unique characteristics associated with this unusual pathogen.

## 1 Introduction

*Rasamsonia argillacea*, formerly classified as *Geosmithia argillacea*, is a thermotolerant filamentous fungus that has emerged as a rare but increasingly recognized opportunistic pathogen. Initially identified as a colonizer of the respiratory tract in patients with cystic fibrosis, it has since been implicated in invasive infections among immunocompromised individuals, particularly those with chronic granulomatous disease (CGD) and hematologic malignancies [1].

The identification and management of *R. argillacea* infections remain particularly challenging. Morphologically, it is easily mistaken for more common molds such as *Penicillium* or *Paecilomyces*, requiring molecular sequencing for definitive diagnosis. *In vitro*, *R. argillacea* exhibits high minimum inhibitory concentrations (MIC) to many azoles, including voriconazole and isavuconazole, limiting therapeutic options. Echinocandins and amphotericin B (AmB) are generally more active *in vitro*, but clinical evidence guiding optimal antifungal therapy remains scarce [2].

We present a unique case of a cerebral abscess caused by *R. argillacea* in a lung transplant recipient without prior fungal colonization. This rare presentation ultimately led to the diagnosis of previously unsuspected chronic granulomatous disease, highlighting the importance of considering rare molds in atypical post-transplant infections and prompting further immunological investigation when standard risk factors are absent.

## 2 Case presentation

A 65-year-old Caucasian woman with end-stage emphysema underwent bilateral lung transplantation one year prior to the presentation. Her past medical history was significant for coronary heart disease, diabetes, hypertension, dyslipidemia and quiescent Crohn's disease. Her immunosuppressive therapy consisted of tacrolimus, prednisone and mycophenolate mofetil. Antimicrobial prophylaxis consisted of trimethoprim/sulfamethoxazole for *Pneumocystis jirovecii* prevention. She presented with generalized weakness, asthenia, night sweats and weight loss over the past 6 weeks and was found to have new bilateral lung nodules on chest X-ray.

Upon admission, investigation revealed multiple bilateral cavitary pulmonary nodules on chest CT (Fig. 1A). Positron emission tomography (PET) performed at day +4 showed hypermetabolic lesions in the lungs, pericardium, bone, subcutaneous, gluteal muscle and lymph nodes (Fig. 1B). Despite the absence of focal neurologic symptoms, cerebral imaging was ordered due to the disseminated process. On day +5, a cerebral magnetic resonance imaging (MRI) revealed a large 6 cm multi-loculated right inferior frontal lesion associated with multiple frontal and occipital lesions surrounded by vasogenic edema (Fig. 1C and D).

**Fig. 1A:**
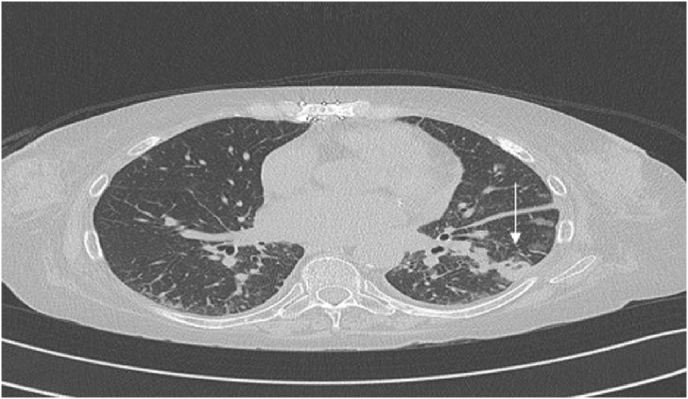
Chest CT showing the left lower lobe condensation (white arrow).

**Fig. 1B:**
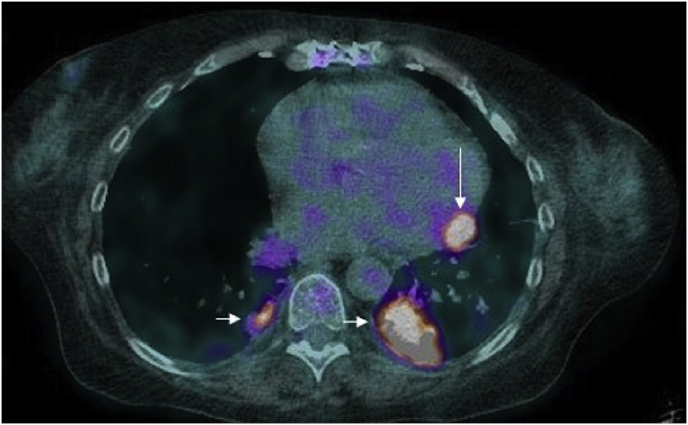
TEP image fusion highlighting hypermetabolic pulmonary lesions (horizontal arrows) and pericardial lesion (vertical arrow). Fig. 1C, Fig. 1DC and D: Left: Brain MRI frontal section showing the multi-loculated right frontal lesion with important vasogenic edema. Right: Axial section of the same lesion in diffusion-weightened imaging (DWI).

**Fig. 1C:**
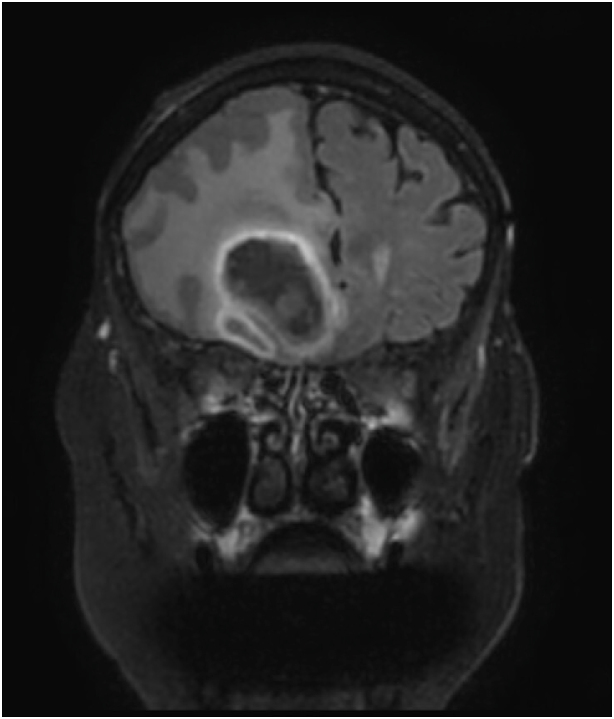
Brain MRI frontal section showing the multi-loculated right frontal lesion with important vasogenic edema.

**Fig. 1D:**
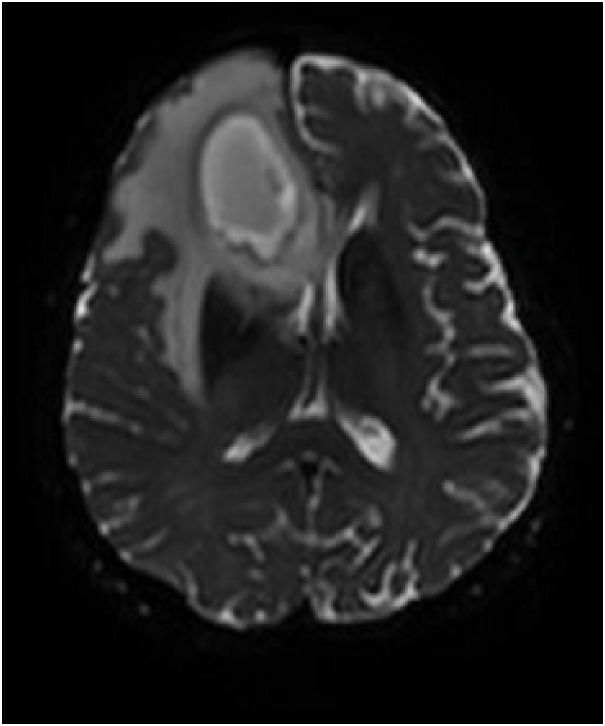
Axial section of the same lesion in diffusion-weightened imaging (DWI).

A cerebral biopsy was immediately performed and recovered 20 ml of purulent material. Empirical intravenous imipenem, trimethoprim-sulfamethoxazole and voriconazole were initiated. On day +10, a beige hyaline mold grew on various fungal culture media (Fig. 2A) and microscopic examination revealed septate hyphae with rough and thin hyaline conidiophores, asymmetrical penicillium-like consisting of metulae in verticils of 3–5 and phialides with long tapering tips producing smooth-walled cylindrical “boxcar-shaped” conidia in chains, that appear ovoid as they age (Fig. 2B). A presumptive identification of *Rasamsonia* sp. was made and subsequently confirmed by ITS/D1D2 rDNA and β-tubulin fungal target sequencing. Antifungal susceptibility testing for *Rasamsonia* reported the following MIC: voriconazole >16 mg/L, isavuconazole >8 mg/L, itraconazole >16 mg/L, posaconazole 2 mg/L, amphotericin B 1 mg/L, caspofungin 0.03 mg/L, micafungin 0.03 mg/L, and anidulafungin at 0.03 mg/L.

**Fig. 2A:**
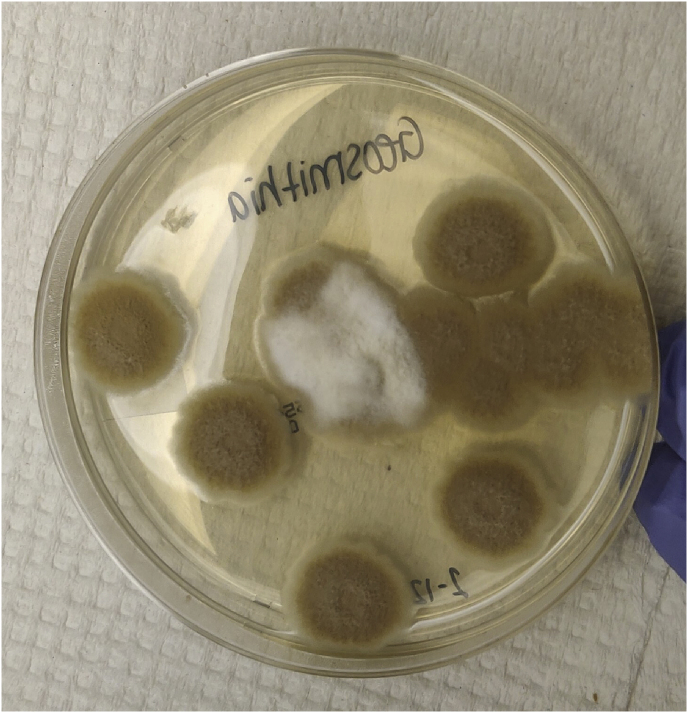
Potato dextrose agar (PDA) displaying brownish and white granular colonies after 7 days at 37 °C. Fig. 2B, Fig. 2AA and B. On the left, potato dextrose agar displaying brownish and white granular colonies after 7 days at 37 °C. On the right, photomicrograph of the lactophenol cotton blue mount (*40x magnification*), showing typical septate hyphae with rough and thin conidiophores, phialides with tapered necks and especially cylindrical “barrel-shaped” conidia (arrow).

**Fig. 2B:**
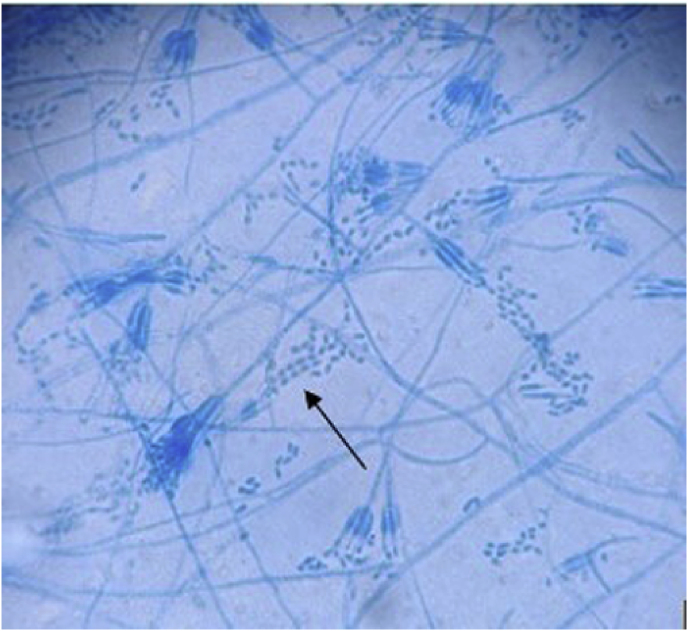
Photomicrograph of the lactophenol cotton blue mount (*40x magnification*), showing typical septate hyphae with rough and thin conidiophores, phialides with tapered necks and especially cylindrical “barrel-shaped” conidia (white arrow).

A transthoracic lung biopsy on day +2 demonstrated septate hyphae with tissue necrosis and subsequent culture grew *Aspergillus udagawae* (formerly known as *Neosartorya udagawae*) with the following susceptibility testing: voriconazole 1mg/L, isavuconazole 0.5 mg/L, itraconazole 0.5 mg/L, posaconazole 0.12 mg/L, amphotericin B 1 mg/L, caspofungin 0.03 mg/L, micafungin <0.0016 mg/L, and anidulafungin <0.0016 mg/L. A skin biopsy of a right scapular nodule on day +21 demonstrated a necrotizing granuloma associated with fungal hyphae and fungal culture grew *Aspergillus nomius* section Flavi with the following susceptibility testing: voriconazole 0.5 mg/L, isavuconazole 0.5 mg/L, itraconazole 0.5 mg/L, posaconazole 0.25 mg/L, amphotericin B 2 mg/L, caspofungin 0.06 mg/L, micafungin 0.06 mg/L, and anidulafungin 0.06 mg/L.

A diagnosis of multiple invasive fungal infections was made and a combination of antifungal therapy with intravenous voriconazole, liposomal amphotericin B (L-AmB) and caspofungin was initiated on day +10. Following antifungal susceptibility results, voriconazole was substituted for posaconazole on day +28, despite lower cerebral penetration of the latter. Throughout treatment, posaconazole therapeutic drug monitoring levels remained between 1.8 and 4.4 mg/L. In addition to antifungal therapy, the patient underwent two surgical drainages (ad day +5 and day +33) for the brain abscess. Despite surgical drainage and antifungal therapy, the patient developed altered mental status due to progressive vasogenic edema and required high dose intravenous dexamethasone. Unfortunately, the patient continued to deteriorate and eventually requested palliative care prior to succumbing on day +82 after admission.

In view of the uncommon and atypical fungal infection, an immunologic workup was performed to exclude a primary immunodeficiency. Neutrophil oxidative burst index was performed twice but remained non-diagnostic due to the presence of a broad distribution shown on dihydrorhodamine flow cytometric assay histogram without an actual peak. Genetic analysis revealed a homozygous deletion encompassing exons 2–11 of *NCF1* gene compatible with a diagnosis of CGD.

## 3 Discussion

Initially described as a thermotolerant *Penicillium* species, *Rasamsonia argillacea* underwent successive reclassifications based on morphological and physiological distinctions, and received its current name in 2011 in honor of mycologist R.A. Samson [3]. The *Rasamsonia* genus comprises twelves species, of which *R. argillacea sensu stricto*, *R. piperina*, *R. aegroticola*, and *R. eburnean* are the most frequently implicated in human disease [4]*. Rasamsonia argillacea* was initially identified as a respiratory tract colonizer in cystic fibrosis patients, though its clinical significance in this context remains debated [3]. Increasingly, it has been recognized as a pathogen with invasive potential, notably in pulmonary and extrapulmonary sites including the central nervous system and cardiovascular system [1].

Routine phenotypic identification of *Rasamsonia* may be challenging as it can often be misidentified as *Penicillium* or *Paecilomyces.* Typically, *Rasamsonia* will grow a tan cream or beige colony with powdery surface within 7 days on standard culture media such as Sabouraud, Potato dextrose (PDA), Cornmeal or Mycosel (MYC) agar [5]. Microscopic examination reveals roughness of the whole fruiting structure and particularly phialides with rectangular « boxcar » shaped conidia [5]. Careful attention to conidia shape may distinguish from similar fungi; *Paecilomyces* and *Penicillium* typically have oval and round shaped conidia respectively and quickly grow at room temperature [6,7]. Matrix-assisted laser desorption ionization–time of flight (MALDI-TOF) shows promising results to overcome *Rasamsonia* morphological identification difficulties [8,9]. *R. argillacea* is now included in both current BioTyper and VITEK MS (V3.2. and later) filamentous IVD databases. However, its diagnostic performance is not perfect and other *Rasamsonia* species are not included in the commercially available clinical databases [10]. DNA sequencing is therefore often required for definite identification [11]. The importance of rapid and accurate identification of *Rasamsonia* is essential to recognize its pathogenic potential, clinical and therapeutic implications.

Management of this potentially lethal infection is particularly challenging. In contrast to the aforementioned molds (*Penicillium* or *Paecilomyces*), *Rasamsonia argillacea* shows elevated MIC to most azoles and is thought to be intrinsically resistant *in vitro* to isavuconazole and voriconazole and may display variable MIC to itraconazole [12,13]. Posaconazole generally displays the lowest MIC amongst azoles. Echinocandins are the most active agent *in vitro* followed by amphotericin B [2,4]. However, no clinical breakpoint or epidemiological cutoff values exist and evidence for management of this type of infection remains sparse. A few successful results have been shown *in vivo* with echinocandins alone (caspofungin or micafungin) or in combination with either L-AmB or posaconazole [2]. Current guidelines therefore recommend using an echinocandin monotherapy or combining it with L-AmB or posaconazole [2]. *In vitro* data also reported low MIC with olorofim and fosmanogepix, respectively first members of the new orotomide and “gepix” class of antifungals, which may be active *in vivo* against *Rasamsonia* [[14], [15], [16]]. There is currently no *in vivo* experience of using these antifungals in the context of *Rasamsonia* infection. Rapid identification leading to appropriate treatment is vital given the high mortality associated with this infection [1]. *Rasamsonia* disease among 23 patients was associated with an overall mortality rate of 39 % and attributable mortality of 17 % [1].

While isolated cases have been described in immunocompetent individuals, *Rasamsonia* infections overwhelmingly occur in the context of well-defined underlying immunosuppressive conditions [1]. CGD is the most common underlying primary immunodeficiency associated with *Rasamsonia* infection [1]. CGD is an inborn error of immunity caused by defects in the phagocyte nicotinamide adenine dinucleotide phosphate (NADPH) oxidase complex resulting in the inability to generate microbicidal reactive oxidants, predisposing to a wide array of infections. Most common pathogens which cause disease include, *Staphylococcus aureus, Serratia marcescens, Burkholderia cepacia complex, Nocardia* spp. and *Aspergillus* spp. While CGD is usually diagnosed in early childhood due to severe infectious or inflammatory complications, other patients have a milder clinical phenotype and are diagnosed later in life as adults. Several factors may contribute to the delayed diagnosis including phenotypic variability leading to misdiagnosis, unusual presentation, unusual pathogen, mild genetic defect, fixation on misdiagnosis and lack of awareness.

Hematologic malignancies, particularly in the setting of hematopoietic stem cell transplantation, represent the second most frequently associated immunodeficiency, often complicated by graft-versus-host disease. The profound and prolonged immunosuppression characteristic of these conditions, including neutropenia and impaired cellular immunity, possibly creates a permissive environment for invasive fungal infections by hyaline molds such as *Rasamsonia* [1,17]. Cystic fibrosis has been identified as a potential risk factor for *Rasamsonia argillacea* isolation, although most reported cases likely reflect colonization rather than true infection [11]. Beyond the three aforementioned conditions, other forms of immunodeficiency, including solid organ transplantation, have not been consistently associated with an increased risk of invasive *Rasamsonia* infection. To date, only two cases of *Rasamsonia* infection have been reported in lung transplant recipients [1,18]. In both cases, invasive *Rasamsonia* disease arose from pretransplant respiratory tract colonization among CF with the same organism. The patient in this current report did not have pretransplant respiratory tract colonization with fungal pathogens. To our knowledge, *de novo Rasamsonia* infection among SOT has not been reported. Thus, the diagnosis of this unusual pathogen in the setting of organ transplantation raised concern for a potentially more profound immunodeficiency beyond that induced by organ transplantation. Immunological assessment confirmed the late diagnosis of CGD which was, until the age of 65, unsuspected. Our case highlights the unusual nature of this microorganism, its multidrug resistance profile and its association with specific immunodeficiency, particularly CGD.

In conclusion, our case is the first case of *Rasamsonia* cerebral abscess in a lung transplant recipient. The unexpected and unusual nature of the microorganism led to the late diagnosis of primary immunodeficiency due to CGD.

## CRediT authorship contribution statement

**Raphaël Schils:** Conceptualization, Data curation, Writing – original draft. **Michaël Desjardins:** Writing – review & editing. **Charles Poirier:** Writing – review & editing. **Hugo Chapdelaine:** Writing – review & editing. **Marie-Pierre Fournier Gosselin:** Writing – review & editing. **Philippe J. Dufresne:** Writing – review & editing. **Me-Linh Luong:** Conceptualization, Methodology, Writing – review & editing.

## Consent

Written informed consent was obtained from the patient or legal guardian(s) for publication of this case report and accompanying images. A copy of the written consent is available for review by the Editor-in-Chief of this journal on request.

## Funding source

There are none.

## Please state any competing interests

Me-Linh Luong is a consultant for Takeda, AVIR and Merck.

Philippe Dufresne has received research grant and financial support from AVIR, Thermo fischer and bioMérieux.
